# Crowd vocal learning induces vocal dialects in bats: Playback of conspecifics shapes fundamental frequency usage by pups

**DOI:** 10.1371/journal.pbio.2002556

**Published:** 2017-10-31

**Authors:** Yosef Prat, Lindsay Azoulay, Roi Dor, Yossi Yovel

**Affiliations:** 1 School of Zoology, Faculty of Life sciences, Tel Aviv University, Tel Aviv, Israel; 2 Sagol School of Neuroscience, Tel Aviv University, Tel Aviv, Israel; Princeton University, United States of America

## Abstract

Vocal learning, the substrate of human language acquisition, has rarely been described in other mammals. Often, group-specific vocal dialects in wild populations provide the main evidence for vocal learning. While social learning is often the most plausible explanation for these intergroup differences, it is usually impossible to exclude other driving factors, such as genetic or ecological backgrounds. Here, we show the formation of dialects through social vocal learning in fruit bats under controlled conditions. We raised 3 groups of pups in conditions mimicking their natural roosts. Namely, pups could hear their mothers' vocalizations but were also exposed to a manipulation playback. The vocalizations in the 3 playbacks mainly differed in their fundamental frequency. From the age of approximately 6 months and onwards, the pups demonstrated distinct dialects, where each group was biased towards its playback. We demonstrate the emergence of dialects through social learning in a mammalian model in a tightly controlled environment. Unlike in the extensively studied case of songbirds where specific tutors are imitated, we demonstrate that bats do not only learn their vocalizations directly from their mothers, but that they are actually influenced by the sounds of the entire crowd. This process, which we term “crowd vocal learning,” might be relevant to many other social animals such as cetaceans and pinnipeds.

## Author summary

The spontaneous acquisition of speech by human infants is considered a keystone of human language, but the ability to reproduce vocalizations acquired by hearing is not commonly described in other mammals. This skill, termed vocal learning, is challenging to study in nonhuman animals since such investigation requires the detection and exclusion of innate developmental effects. The recognition of vocal dialects among different populations can open a window on the vocal learning abilities of animals, but such findings in the wild may reflect genetic or ecological differences between groups rather than the learning of group-specific vocal behavior. In this study, we used a playback-based lab experiment to induce vocal dialects in fruit bat pups. By exposing groups of pups to different playbacks of conspecific calls, we could establish separate dialects, demonstrating the vocal learning skill of these bats. Furthermore, while songbirds, for instance, learn their songs directly from a specific tutor, our bats showed the ability to pick up vocal variations from the surrounding crowd, without direct interaction with any given tutor.

## Introduction

**Fig 1:**
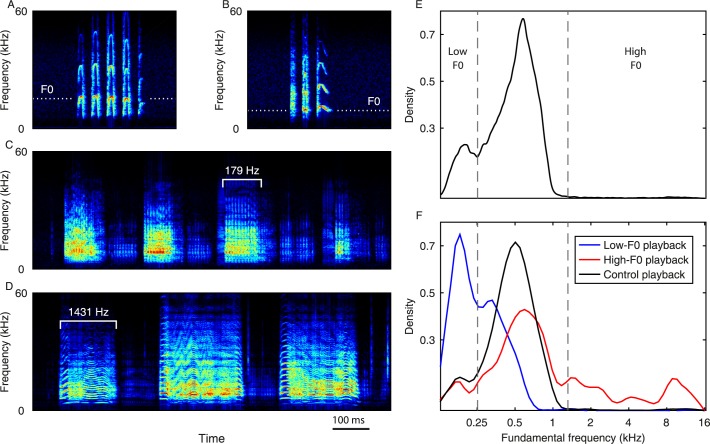
Egyptian fruit bat vocalizations. **(A)** Isolation call—produced by newborn pups. **(B)** A modified isolation call—the first non-isolation social calls of pups (appearing around the age of 20–40 days). White dotted lines in (A) and (B) mark the F0; notice the drop in F0. **(C)** Adult multisyllabic vocalization. One example, out of a diverse repertoire, containing a low F0 call (179 Hz). **(D)** Another example of an adult vocalization containing a high F0 call (1,431 Hz). Notice how in the first call in the sequence the harmonics are clearly separable due to the high fundamental. **(E)** The distribution of adult calls’ F0. Calls with F0 lower than 250 Hz were designated by us as “Low-F0,” and calls with F0 higher than 1,315 Hz were designated as “High-F0” (see Materials and methods for details). **(F)** The distribution of F0 among the 3 playbacks: Blue represents Low-F0 playback, red represents High-F0 playback, and black represents Control playback. Distributions are plotted as smoothed and normalized histograms. Numeric data for (E) and (F) are given in S1 Data. F0, fundamental frequency.

Vocal learning, the ability to learn to produce vocalizations by hearing, is essential in human language acquisition, but only a few other mammals appear to possess this capability [1–8]. Some indications for the existence of vocal learning in nonhuman animals arise from the observation of group-specific vocal dialects in wild populations [9–11]. Such vocal variations can indeed stem from vocal learning of typical vocalizations by members of the group; however, it is usually impossible to completely exclude other explanations for the appearance of vocal differences between populations [12]. For instance, genetic variations may lead to unique vocal patterns, and environmental constraints may induce specific usage of vocalizations. Studies of several species of bats have indicated their vocal learning ability [4]. Early studies suggested that *Phyllostomus discolor* pups adapt their isolation calls to their mothers’ directive calls [13], and *P*. *hastatus* females were shown to maintain a group-specific foraging call through vocal learning [14]. Geographic variations in vocalizations of these 2 species were also observed [15,16], though genetic and environmental factors were not excluded as possible contributors to these apparent dialects. In another bat species (*Saccopteryx bilineata)* that is an important model for vocal learning, pups have been shown to learn territorial songs from adult male tutors [17] and to engage in vocal babbling behavior [18]. In a previous study [19], we showed that depriving Egyptian fruit bat (*Rousettus aegyptiacus*) pups from hearing adults delays their vocal ontogeny. Yet we also found that these isolated pups eventually catch up with their control counterparts. Moreover, we have not shown plasticity in the vocal ontogeny of non-isolated pups. The Egyptian fruit bat is an extremely social and vocal mammal, living in colonies of dozens to thousands of individuals. In the wild, these bats are exposed to extensive vocal communication throughout their entire lives. A typical vocalization of this species is composed of a sequence of multiharmonic calls (Fig 1; see Materials and methods for details). The fundamental frequency (F0) in newborn pup isolation calls is high (ca. 8–15 kHz) (Fig 1A), and it gradually decreases to ca. 0.2–1.2 kHz in adults (Fig 1B–1D). We have previously shown that this process involves vocal learning [19].

A fruit bat pup is mostly exposed to adult vocalizations when in the roost. In this situation, the pup continuously hears countless vocalizations coming from the surrounding darkness and has very little, if any, interaction with most of the vocalizing individuals. It is therefore exposed to a cacophony of fruit bat vocalizations, only a slight minority of which are emitted by its mother or by nearby roostmates. In this study, we therefore set to examine whether the vocal communication of pups that grow up in such an environment is shaped by the individuals that they directly interact with or by the background vocalizations they are “passively” exposed to. We raised pups in conditions that mimic the natural acoustic conditions of a dark fruit bat cave and observed the establishment of vocal dialects through vocal learning of the entire “crowd” in the artificial cave.

## Results

We caught pregnant female Egyptian fruit bats in wild roosts in central Israel. The bats were then randomly assigned to 3 identical and acoustically isolated chambers. Each female gave birth to a single pup in these chambers (resulting in 3 groups of 5, 5, and 4 pups). The mothers were released a few weeks after the pups were weaned (at the age of ca. 14 weeks). In each of the 3 chambers, a playback of conspecific vocalizations was constantly played from day 1 and for a full year (see Materials and methods). The playback intensity and frequency mimicked the vocalizations of ca. 100–200 adults. The pups were thus exposed to a situation similar to a natural roost, hearing their mothers' vocalizations embedded within the noise created by a crowd of hundreds of bats.

The playbacks were sampled from a set of thousands of agonistic vocalizations previously recorded in the same setup. Agonistic calls constitute almost all of the vocalizations emitted in the roost by this species [20]. They are elicited as a response to unsolicited physical contact and are characterized by a typical range of acoustic features (S1 Fig). We chose to vary the F0 of the calls after observing (in [19]) that this is a feature that is strongly influenced by exposure to adult vocalizations. According to the distribution of F0 across bat calls, we defined 3 groups of calls: low-fundamental calls (Low-F0, with F0 lower than 250 Hz), high-fundamental calls (High-F0, with F0 higher than 1,315 Hz), and intermediate-fundamental calls (the rest and the majority of calls, see Fig 1E and Materials and methods for details). Playbacks were assigned to each experimental group according to their F0 content. The control group (*n* = 5) was exposed to playbacks randomly sampled from the previously recorded repertoire with an average F0 of 564 Hz (1% High-F0 and 11% Low-F0 calls, black line in Fig 1F). The Low-F0 group (*n* = 5) was exposed to playbacks with an average F0 of 303 Hz (0.2% High-F0 and 52% Low-F0 calls, blue line in Fig 1F). The High-F0 group (*n* = 4) was exposed to playbacks with an average F0 of 1,871 Hz (26% High-F0 and 9% Low-F0 calls, red line in Fig 1F). Note that the High-F0 group was exposed to a playback that was highly unnatural in 2 ways: 1) it contained approximately 26 times more high-frequency calls than the typical adult repertoire (the Low-F0 playback only contained approximately 4.5 times more Low-F0 calls), and 2) pup vocal ontogeny is typically characterized by decreasing the call fundamental, while this playback aimed to drive the pups in the opposite direction.

We therefore expected that the High-F0 playback would be more difficult to mimic than the Low-F0 playback.

The pups were housed in their birth chambers for the entire experiment period (approximately 1 year), except for during the recording sessions, and the playbacks were constantly played in these birth chambers throughout the year of the experiment. The pups were recorded 4 times during the experiment, at the ages of 12–18 weeks, 31–35 weeks, 40–43 weeks, and 48–51 weeks. To ensure identical recording conditions, the recordings were performed in a fourth identical acoustic chamber. Each group was moved to the recording chamber for a few days in a rotating manner throughout each recording session (which therefore lasted approximately 1 month). As expected, all recorded vocalizations were agonistic (elicited as a response to unsolicited physical contact), and no behavioral differences were observed between the groups.

**Fig 2:**
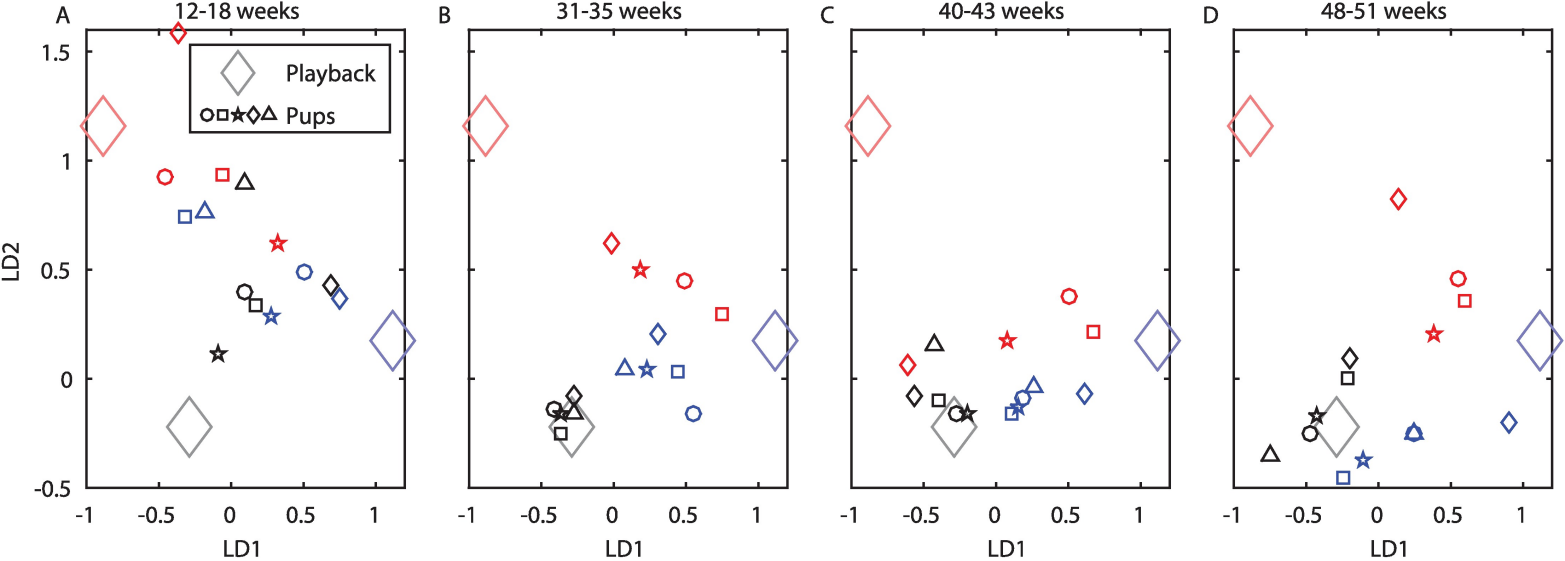
Dialect formation. Acoustic parameters of bat calls during their development, at the ages of **(A)** 12–18, **(B)** 31–35, **(C)** 40–43, and **(D)** 48–51 weeks. The average for each bat (small symbols) and for each playback (large diamond) is presented. Blue represents the Low-F0 group (*n* = 5), red represents the High-F0 group (*n* = 4), and black represents the control group (*n* = 5). The axes were obtained by an LDA of the playbacks (see text for details), where the features that contributed the most were the F0, the energy entropy, and the spectral centroid (S1 Table). The axes of the 4 panels are the same as they were determined by the playbacks, and the playback location is constant in all stages. Numeric data are given in S1 Data. F0, fundamental frequency; LD1, first linear discriminant; LD2, second linear discriminant; LDA, linear discriminant analysis.

The pups in the 3 treatments developed 3 distinct vocal dialects over time. In order to quantify the acoustic differences between the pups and test for any relation to the playbacks, we first calculated a set of 7 acoustic features for each recorded call and for each of the playback calls (see Materials and methods). Using these features, we performed a linear discriminant analysis (LDA) on the calls of the 3 playbacks to obtain the 2 axes that best separated the playbacks (S1 Table). We then projected the recorded pup vocalizations on these 2 axes (Fig 2; see Materials and methods for details and S2 Table for number of analyzed calls). At a very young age (after 12–18 weeks of exposure to the playback), a large variability was observed with no significant distinction between the groups (Fig 2A; permutation test for linear discriminability: *p* = 0.09), though some discrepancy between them may have already been present. When the pups matured, the groups became acoustically significantly separable (Fig 2B–2D; permutation tests for linear discriminability: *p* = 3.2 × 10^−5^, *p* = 0.0075, and *p* = 5.6 × 10^−5^ at the ages of 31–35, 40–43, and 48–51 weeks, respectively). These findings present the formation of 3 dialects in the lab and suggest a connection between the established dialects and the auditory experience (as explained below).

Importantly, discriminant analysis is typically used on the “experimental data” (in our case, the pups' vocalizations) to examine separation; however, in the current analysis, the axes presented in Fig 2 were chosen to discriminate between the “treatments” (i.e., the playbacks) and not between the pup vocalizations. This means that we did not deliberately project the data on the dimensions that separated the pups best but rather on the predefined dimensions that best separate the stimulus they were exposed to. Therefore, the axes of all of the panels in Fig 2 are the same (as they were determined by the playbacks). The fact that the pup calls were clustered into 3 distinct groups along these treatment-axes strongly suggests that they were influenced by the playbacks. The acoustic features that mainly contributed to these 2 separating axes include the F0, the energy entropy, and the spectral centroid (S1 Table). It is also important to note that the pups were recorded in an environment with no playbacks (in the recording chamber); thus, they were recorded when interacting with each other after they had assimilated the conspecific vocalizations heard in their home chambers.

**Fig 3:**
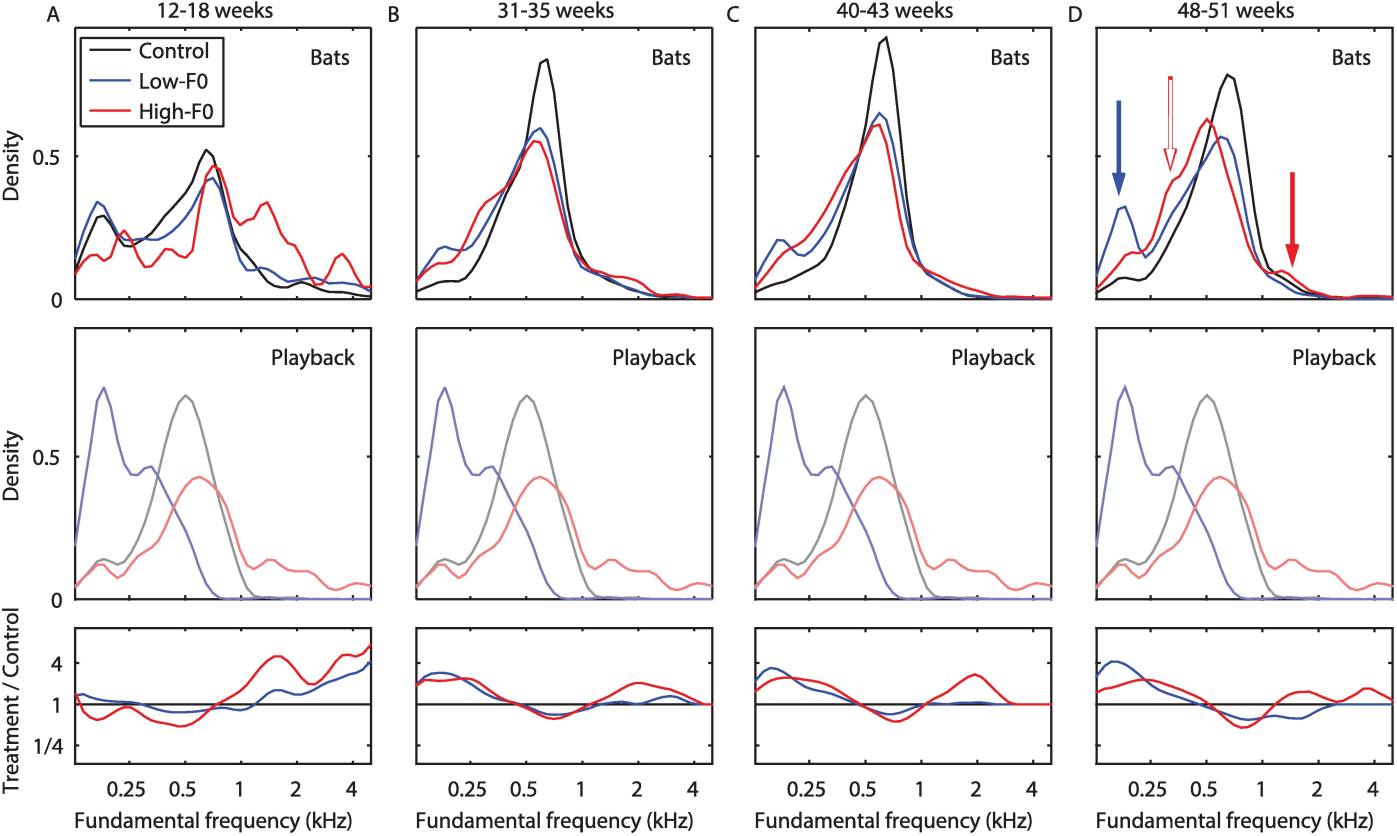
The playbacks direct the dialect formation. The distribution of F0 usage by the 3 groups (top panels): control (black), Low-F0 (blue), and High-F0 (red). The 4 recording sessions are presented as 4 columns: **(A)** 12–18 weeks, **(B)** 31–35 weeks, **(C)** 40–43 weeks, and **(D)** 48–51 weeks. The presented distribution is the average for all pups in the group (see S2 Fig for the usage distribution of each pup separately). For comparison, the middle panels depict the F0 distribution in the playback of each group, as depicted in Fig 1F (the same for all recording sessions). The lower panels show the ratio of each distribution (in the upper panels) to that of the control group (in log-scale). The filled arrows in the upper panel of (D) show the main effect of the playback in each group, while the empty arrow shows an unexpected increase in the Low-F0 usage by the High-F0 group. Numeric data are given in S1 Data. F0, fundamental frequency.

To directly test the effects of the playbacks on the pups, we compared the acoustic parameter that we directly manipulated—i.e., the use of different F0 in each of the groups (Fig 3, S2 Fig). The F0 distributions in the Low-F0 group and the High-F0 group were indeed biased according to their respective playbacks (linear mixed models; A model for usage of Low-F0 calls: significant difference between the groups - *p* = 0.0004, post-hoc test for difference between the Low-F0 and control groups - *p* = 0.0001; A model for usage of High-F0 calls: significant difference between the groups - *p* = 0.0008, post-hoc test for difference between the High-F0 and control groups - *p* = 0.0002; see Materials and methods for details). All 3 groups mostly used calls with F0 around the peak of the control distribution (approximately 600 Hz), suggesting an innate preference (see Discussion). However, the pups in the Low-F0 group used significantly more low-fundamental (i.e., lower than 250 Hz) calls than the control group from the age of ca. 31 weeks onward, in accordance with the playback they were exposed to (blue line and blue arrow in Fig 3, Mann–Whitney U test: *p* = 0.004, *p* = 0.004, and *p* = 0.016 in the second, third, and fourth recording sessions, respectively; S3J–S3L Fig). Similarly, the High-F0 group used significantly more high-fundamental (i.e., higher than 1,315 Hz) calls than the control group, at least until the age of ca. 43 weeks in accordance with the playback they were exposed to (red line and red arrow in Fig 3; Mann–Whitney U test: *p* = 0.032, *p* = 0.032 in the second and third recording sessions, respectively; S3B and S3C Fig). Because of their small absolute number, the use of High-F0 calls by this group can be better seen when examining the ratio between the distribution of the High-F0 and the control groups (Fig 3, bottom row).

We controlled for the possibility that the dialects we observed resulted from physiological or genetic differences. We verified that the bats within each group were not more genetically related to each other than to the bats in the other groups or to bats in the general population, i.e., the intragroup relatedness did not significantly differ from the intergroup relatedness or the general population relatedness (see Materials and methods). We also verified that there was no significant difference in F0 usage between males and females (S3 Table, S4 Table) and that there was no correlation between body size (estimated by body weight) and F0 usage in any group at any recording session (S4 Table).

## Discussion

This study adds substantial evidence for the importance of vocal learning in the ontogeny of bat vocal communication. The highly controlled playback experiments that we performed excluded possible biasing factors such as differences in the ecological, developmental, or genetic backgrounds of the subjects or even differences in the recording conditions, all of which might lead to false reports of vocal learning. It is important to note that, in the wild, as well as in our setup, bats are exposed to an immense amount of vocalizations produced by conspecifics in the dark. Thus, young pups hear conspecifics that do not directly interact with them to an extent that quantitatively overshadows the vocalizations produced by their mothers or immediate neighbors. Accordingly, we found that our pups presented a “crowd vocal learning” phenomenon, where their vocal repertoire was shaped by the complete repertoire they heard in their colony (mainly governed by our playbacks) and not only by the vocalizations of a single tutor (e.g., their parents) as is mostly discussed in the songbird literature [21]. Vocal learning is often assumed to include imitation [1] or at least social reinforcement of specific vocalizations [8]. The bats in our study did not interact with their models and hence were not subject to reinforcement, and we cannot assert that they imitated specific calls. It may be in line with recent views, which dispute the dichotomous definition of (presence or absence of) vocal learning abilities and rather find varying levels of this skill among different species [22]. Furthermore, when syllables are not readily categorized into specific types, as in the case of fruit bat vocalizations [20], it might be more difficult to identify imitation than when clear syllable types are recognized (as in the case of many birdsongs). Yet the bat crowd vocal learning demonstrates some degree of imitation, with an apparent tendency to social conformity. We hypothesize that such crowd vocal learning may be employed by other species that are exposed to many vocalizations of conspecifics without directly interacting with them. Such auditory exposure occurs, for instance, in many cetaceans, whose calls travel very long distances, or in congregating species such as pinnipeds and some sea birds (in which vocal learning has so far not been described).

Several aspects of the behavior of the High-F0 group suggest that innate preferences also play a role in vocal ontogeny: 1) The bats have not adopted calls with F0 above 2 kHz, although these were abundant in the playback. Such high F0 calls characterize subadults and are very rarely emitted by adults, and 2) They reduced the use of high F0 calls when reaching sexual adulthood. At the age of 43 weeks (approximately 300 days), the bats are already mature, and the use of high-fundamental calls at this age is extremely rare in fruit bats (possibly due to physical constraints). Hence, it seems that a bias that is related to the animal’s physiology overrides learning of too-high-fundamental calls after a certain age (High-F0 group, Mann–Whitney U test: *p* = 0.14 in the fourth recording session; S3D Fig).

Note also that the High-F0 bats also included more low F0 calls in their repertoire relative to the controls (red outlined arrow, Fig 3). We can only hypothesize that this was due to their lesser exposure to calls around the control peak (approximately 600 Hz). Importantly, even if the High-F0 bats reduced the excess of high-frequency calls in their repertoire towards the end of the year, they still exhibited their unique vocal dialect that was also driven by additional acoustic properties. This can be learnt from the forming of separable groups in the time period of the last recordings (Fig 2B–2D, note that the probability of getting a separable group by chance is extremely low; see for example 4 random permutations in S4 Fig and exact *p*-values above). One acoustic feature that contributed to the unique dialect of the High-F0 group was the energy entropy (S5 Fig; also conforming to the LDA analysis in S1 Table).

To conclude, in a tightly controlled acoustic environment, we observed the formation of vocal dialects as a result of crowd vocal learning. When such dialects are found in the wild, it is often difficult to exclude nonsocial factors, but in this study, the pups were raised and recorded in identical settings except for the playback they heard. Notably, shared intragroup behaviors acquired and transmitted through social learning are generally referred to as culture [12,23]. Furthermore, evidence for nonhuman culture is occasionally based on learned vocal behaviors of birds [24–26] and mammals [27,28], with specific emphasis on vocal dialect variations between wild populations [29–31]. In our study, though pups did not directly learn from conspecifics, they were actually exposed to a conspecific stimulus that is very similar to that available to them in the wild (i.e., a stimulus that includes sound without vision or touch). Hence, our results demonstrate the assimilation of shared behavioral phenotypes, which were acquired by social vocal learning from a conspecific stimulus and thus might be considered as in-lab establishment of (vocal) culture in a mammalian model.

## Materials and methods

### Animal capture and care

Adult, heavily pregnant female bats (*R*. *aegyptiacus*) were captured in 2 wild roosts in central Israel and were randomly mixed. The bats were kept in 3 identical acoustic chambers (length: 190 cm; width: 90 cm; height: 82 cm) large enough to allow flight and fed with a variety of fruit ad lib. The light/dark regime was 12 h/12 h. The bats were randomly assigned to 3 groups, each housed in 1 chamber: 5 bats in the High-F0 group, 5 bats in the Low-F0 group, and 5 bats in the control group. All bats gave birth inside the chambers. One pup of the High-F0 group and 1 pup of the control group died few days after birth. Subsequently, 1 mother with a pup approximately 1.5 months old (caught in the wild roost) was added to the control group when the pups were ca. 1.5 months old.

### Ethics statement

All experiments were reviewed and approved by the Animal Care Committee of Tel Aviv University (Number L-13-016) and were performed in accordance with its regulations and guidelines regarding the care and use of animals for experimental procedures. The use of bats was approved by the Israeli National Park Authority.

### Playback

In previous studies in this exact setup, we have recorded hundreds of thousands of bat vocalizations. Examining the distribution of the F0 among the recorded adult and subadult vocalizations (Fig 1E), we defined 2 extreme groups of calls—High-F0 (above 1,315 Hz, 2 SD above the mean) and Low-F0 (below 250 Hz, which is the minimum between the 2 modes in the bimodal distribution, 1.1 SD below mean). For the playbacks (Fig 1F), we sampled the original dataset with 2 biased samples: one containing a high proportion of Low-F0 calls, which was played to the Low-F0 group, and one containing a high proportion of High-F0 calls (including subadult vocalizations), which was played to the High-F0 group. For the control group, we used a random sample (see diamond shapes in Fig 2; see also S3 Fig and lines in the middle row of Fig 3 for the F0 content of the playbacks). We used raw recordings (audio files) without any editing to keep the stimulus as natural as possible. All in all, 105, 227, and 191 different recordings were included in the High-F0, Low-F0, and control playbacks, respectively (each group was exposed to the same number of played recordings during the entire experiment period, where each recording included a sequence of calls and represented a full vocal interaction that was recorded between adult bats; see below). The playback vocalizations were played around the clock with a timing distribution mimicking the natural vocal behavior of this species, where many of the vocalizations are emitted at dawn and dusk and more vocalizations are emitted during the night than during the day [20]. In each playback event, 1 vocalization (a raw recording of a sequence of calls) was selected randomly for each group, and these vocalizations were played concurrently in their corresponding chambers, i.e., the playbacks were played in a random, nonrepeating order. The rate of the playbacks was 14,057 call-sequences (i.e., recordings) per day and was the same in all 3 groups. Because not all sequences had the same number of calls, the groups heard 69,931, 48,651, and 129,715 calls per day on average for the Low-F0, High-F0, and control groups, respectively (to clarify the difference between a recording and a call, see Fig 1C, where a recording with 4 calls is shown, and Fig 1D, depicting a recording with 3 calls). These might seem like large differences, but even in the treatment with the fewest calls (i.e., 48,651 calls per day), the pups were exposed to a playback rate that was approximately 16 times higher than the calling rate of 5 adult bats [20]. Thus, pups heard (at least) 16–30 times more playback vocalizations per day than the vocalizations produced by their mothers during the first 14 weeks of the experiment (when the mothers were still present).

### Recording of pup vocalizations

We recorded the pups’ vocalizations in 4 recording sessions, when the pups were at the ages of 12–18 weeks, 31–35 weeks, 40–43 weeks, and 48–51 weeks. All ages are reported with an accuracy of ±15 days. During a recording session, each group of pups was transferred into a recording chamber, which was similar to the housing chambers. All pups in a group were transferred together (except for part of the first recording session in which the pups were recorded in triplets; see S5 Table), recorded for 1–5 days, and returned to their home chamber. This transfer was repeated for each group in rotation until the end of the recording session, which lasted for 21–45 days, resulting in all groups being recorded for approximately the same time and no more than a few days apart (see S5 Table for the detailed schedule). The recording chamber was continuously monitored with IR-sensitive cameras and omnidirectional electret ultrasound microphones (Avisoft-Bioacoustics Knowles FG-O; 2 microphones in a cage, 1 in each side of the cage). Audio was sampled using Avisoft-Bioacoustics UltraSoundGate 1216H A/D converter with a sampling rate of 250 kHz. Raw audio recordings were automatically segmented and filtered for noises and echolocation clicks, leaving only bat social communication calls (see [19] for details of this process). The video was synchronized to the audio, resulting in a short movie accompanying each audio recording. Videos were then analyzed by L.A., who identified the emitter of each call. The bats were individually marked using fur bleaching. An emitter bat was recognized by its mouth movements, and 2–3 cameras could be used to verify a distinct assignment. If there was any doubt regarding the emitter's identity, we excluded the vocalization from the analysis.

### Data analysis and statistics

Social vocalizations of *R*. *aegyptiacus* are composed of sequences of separated calls (in our analysis, we regarded a call as a vocalized segment of a duration of at least 20 ms that is separated by at least 4 ms of silence from other vocalized segments). The vocal sequences commonly contain between 1 to 20 calls, with an average length of 2.7 calls (±2.6, SD) per sequence (see examples in Fig 1C and 1D) and an average duration of 119.1 ms (±69.3 ms, SD) per call. These calls are typically broadband (with 90% of the energy spread between approximately 3–45 kHz), generally harmonic squawks, with an average F0 of 544 Hz for an adult bat (F0 for a single call was defined as the geometric mean of the F0 content in that call). The calls are not readily clustered into different acoustic syllables (in the past, we have tested many more features than were used in this paper). They rather appear to rest on an acoustic continuum (see S1 Fig for a description of different acoustic features across the repertoire). They can thus all be considered as variations of one large “acoustic cloud” of agonistic calls. For each call, 7 acoustic features were extracted: log F0, Shannon entropy of the power spectrum, Wiener entropy, spectral centroid, frequency with peak energy, amplitude entropy, and duration. The features were measured with a sliding window of 20 ms (19 ms overlap) and were averaged for each call (except for the duration, which was measured for the entire call). The F0 was calculated using the YIN algorithm [32]. This processing was computed over all recorded calls as well as all playback calls.

We first examined the differences between the groups and their relation to the playbacks using LDA (Fig 2). To this end, we performed an LDA on the features extracted from the 3 playbacks, obtaining the 2 discriminant functions (a projection of the 7 acoustic features onto a new 2-dimensional space, S1 Table) that best discriminate between the playbacks. We then plotted the average of the calls of each pup in each recording session in these new 2 dimensions. The features were scaled prior to the application of the LDA by subtracting the mean and dividing by the SD, for both the playbacks and the pup vocalizations. The separation between the groups, which is clearly visible from the second recording session onwards, was evaluated for statistical significance (using permutations) as follows: For each recording session (each panel in Fig 2), we tested the linear separation between the group, i.e., how many pups are correctly assigned to their group if straight lines are drawn to best separate the groups (this was done using a second LDA applied to obtain the separation significance). We then tested all possible permutations of group assignments for the pups, keeping the number of pups in each group constant, and computed an exact *p*-value (correct assignments in best separation: 10/14, 14/14, 12/14, and 14/14, with *p*-values: 0.09, 3.2 × 10^−5^, 0.0075, and 5.6 × 10^−5^, for recording sessions 1–4, respectively). To control for possible sex biases (i.e., differences between males and females), we repeated these permutations while also keeping the male/female compositions of the groups, obtaining similar results (*p* = 0.1, *p* = 6.8 × 10^−5^, *p* = 0.0076, and *p* = 2×10^−4^, for recording sessions 1–4, respectively).

In order to assess the statistical significance of the use of different F0 (S3 Fig), we performed a mixed linear model analysis, testing the effect of the group on the development of Low-F0 usage or High-F0 usage. We also tested for a possible effect of the sex of the pups (including it in the models) and found no such significant effect (see S3 Table). After finding an overall group effect, we used 1-tailed Mann–Whitney U tests to demonstrate the differences between the manipulation groups and the control group at each recording session (S2 Fig). The mixed model analysis was performed in SPSS. All other processing and the analysis of the data were performed using Matlab 8.

### Genetic analysis

#### Sample collection

3-mm diameter wing punch was sampled from each of 11 individuals (2 pups from the Low-F0 group and 1 pup from the control group died after the recordings were completed but before the samples were taken a few months after the end of the experiment). Punches per individual were preserved in molecular grade 100% ethanol and frozen at −80°F. Wing tissues were obtained using sterile, disposable 3-mm skin biopsy punches. One biopsy punch was used per individual, and samples were taken from regions of the wing that were far enough from major blood vessels and the edge of the wing to avoid tearing.

#### Molecular methods and genetic analyses

Genomic DNA was extracted using DNAeasy tissue Extraction kit (Qiagen, Valencia, California). Samples were genotyped at 10 microsatellite marker loci developed for *R*. *madagascariensis* or *R*. *leschenaulti* using described conditions [33,34]. Amplified products were visualized on an ABI 3100 genetic analyzer. Allele size scoring was performed using GeneMarker v2.6.7 (SoftGenetics, LLC), verified and amended by eye. We examined the deviation from Hardy–Weinberg equilibrium (HWE) and the presence of null alleles using the software Cervus v3.0.7 [35]. Pairwise relatedness was calculated using the package 'related' in R [36]. Microsatellite markers were polymorphic (mean allele number per locus 5.5, range: 2–7), did not deviate from HWE, and had low level of null alleles (< 15%).

#### Genetic results

Relatedness estimates were qualitatively similar across the various estimators used. Using Wang (2002) estimator [37], the relatedness estimate within groups was *r* = −0.064 ± 0.064 (mean ± se) and between groups was *r* = −0.066 ± 0.032 (mean ± se), confirming that relatedness within groups was not different than between groups.

The numerical data used in all figures are included in S1 Data.

## Supporting information

S1 Fig Acoustic features of *R*. *aegyptiacus* agonistic vocal repertoire. (**A**) Energy entropy and peak-frequency; (**B**) Fundamental frequency (F0) and spectral centroid; (**C**) Spectral entropy and Wiener entropy; (**D**) Duration and energy entropy.
(PDF)

S2 Fig The playbacks direct the dialect formation (F0 distribution for each pup). The distribution of fundamental frequency (F0) usage by each of the pups in the three groups: *High-F0* (**A-D**), control (**E-H**), and *Low-F0* (**I-L**). Each pup is plotted with a different line pattern. The four recording sessions are presented, at the ages of (**A,E,I)** 12–18, **(B,F,J)** 31–35, **(C,G,K)** 40–43, and **(D,H,L)** 48–51 weeks.
(PDF)

S3 Fig Proportion of High, Low, and intermediate F0 usage. The proportion of High-F0 calls (**A-D**), intermediate F0 calls (**E-H**), and Low-F0 calls (**I-L**) in the vocalizations of the *High-F0* group (red), *Low-F0* group (blue), and control group (black). The dashed lines in **(A,E,I)** show the relevant proportion of calls in the **playbacks** of the *High-F0* group (red), *Low-F0* group (blue), and control group (black). The four recording sessions are presented, at the ages of **(A,E,I)** 12–18, **(B,F,J)** 31–35, **(C,G,K)** 40–43, and **(D,H,L)** 48–51 weeks. Both *High-F0* and *Low-F0* groups are significantly different than the control group in panels **B,C,F,G,H,J,K,L** One-tailed Mann-Whitney-U test, *p* ≤ 0.03 (see Methods for complete statistical analysis).
(PDF)

S4 Fig Examples of four permutations of group labels. Each row is parallel to Fig 2, but the group identities of the pups were permuted. Four random permutations are shown (**A-D**, **E-H**, **I-L**, and **M-P**). The presented 4 permutations are just the first 4 that were sampled (we did not choose specific permutations), illustrating how difficult it is to get separation by chance (see text for exact p-values). The average for each bat (small symbols) and for each playback (large diamond) is presented. Blue–*Low-F0* group (*n* = 5), red–*High-F0* group (*n* = 4), and black–control group (*n* = 5). The axes were obtained by an LDA of the playbacks (see text for details). Numeric data are given in S1 Data (‘Fig 2‘ sheet)–while group identities were randomly permutated for this figure.
(PDF)

S5 Fig Dialect formation in the *High-F0* group also explained by the energy entropy. The distribution of energy entropy (Shannon entropy of the amplitude) in the *High-F0* and control groups (top panels): control (black), *High-F0* (red). The four recording sessions are presented as four columns: **(A)** 12–18 weeks, **(B)** 31–35 weeks, **(C)** 40–43 weeks, and **(D)** 48–51 weeks. The presented distribution is the average for all pups in the group. For comparison, the bottom panels depict the energy entropy distribution in the playback of both groups.
(PDF)

S1 Table Linear discriminant analysis of the playbacks. The standardized coefficients (i.e. feature’s contribution to the discrimination in each discriminant function) and the correlation of each feature with the two linear discriminant (LD) functions. Top three are in bold. As expected by the selection of the playbacks the fundamental frequency (F0) has the major effect. The error rate in the playback discrimination was 26.4%.
(PDF)

S2 Table Number of calls analyzed for each pup in each recording session. (PDF)

S3 Table Linear mixed models for the usage of Low-F0 and High-F0 calls. Two models are presented (one for Low-F0 and one for High-F0 calls). The models included the age of the pups (four time points, i.e. recording sessions), the sex of the pups, and the group (i.e. Control, *Low-F0* group, or *High-F0* group). The second table of each test contains post-hoc pairwise comparisons of estimated marginal means (Bonferroni-adjusted for multiple comparisons). The p-values of factors that significantly influenced the calls are marked in bold. Insignificant interactions were removed and the models were recomputed. Analysis conducted in SPSS.
(PDF)

S4 Table Similarity between males and females in F0 usage, and no correlation between body-weight and F0 usage. For each recording session, p-values (with statistic) are depicted for Mann-Whitney-U test for differences between the sexes in high-F0 content, mean F0 production, and low-F0 content. For each recording session, p-values (with statistic) are depicted for Spearman correlation tests for correlations between body weight and high-F0 content, mean F0 production, and low-F0 content. All tests indicated insignificant relations. For each recording session, p-values (with statistic) for body-weight differences between the three experimental groups (Kruskal-Wallis test) show that there was no significant weight difference between the groups.
(PDF)

S5 Table Recording schedule. * Recording rotations with only 3 pups at a time, each rotation with a different individual composition.
(PDF)

S1 Data The numerical data used in the figures. (XLSX)

## Abbreviations

F0: fundamental frequency
HWE: Hardy–Weinberg equilibrium
LDA: linear discriminant analysis
