# Supplementary figures and images for: Crowd vocal learning induces vocal dialects in bats: Playback of conspecifics shapes fundamental frequency usage by pups
Source: PLoS Biol. 2017 Oct 31;15(10):e2002556. doi: 10.1371/journal.pbio.2002556 (PMC5663327; doi:10.1371/journal.pbio.2002556)

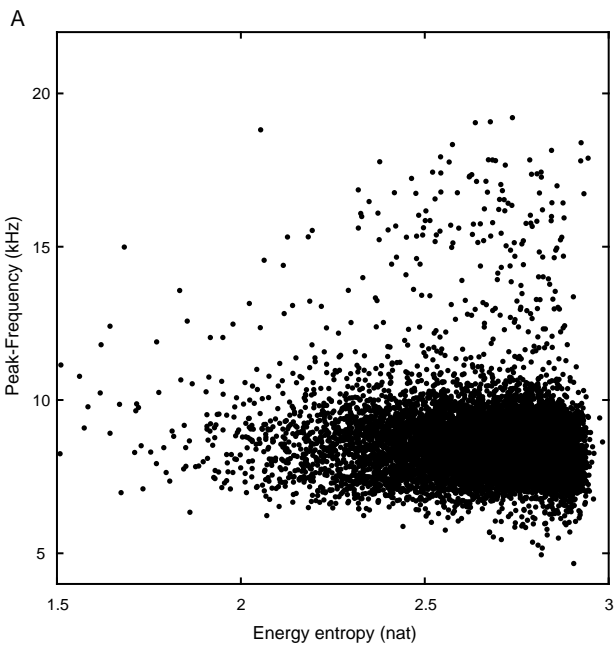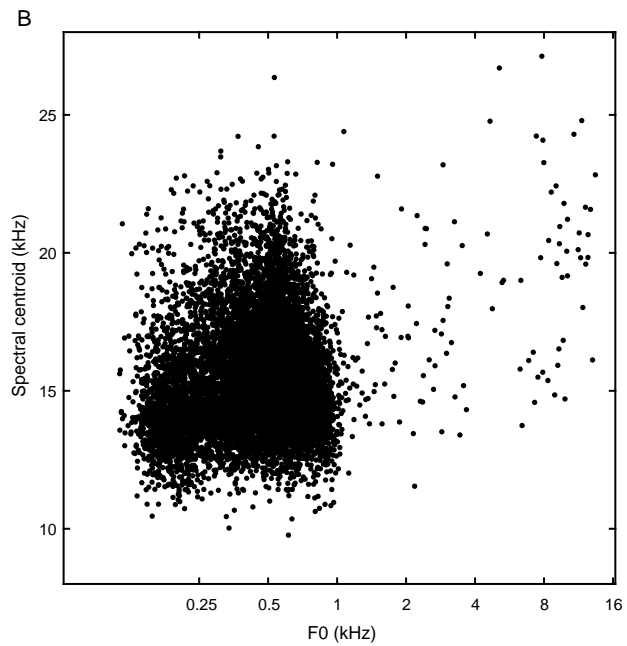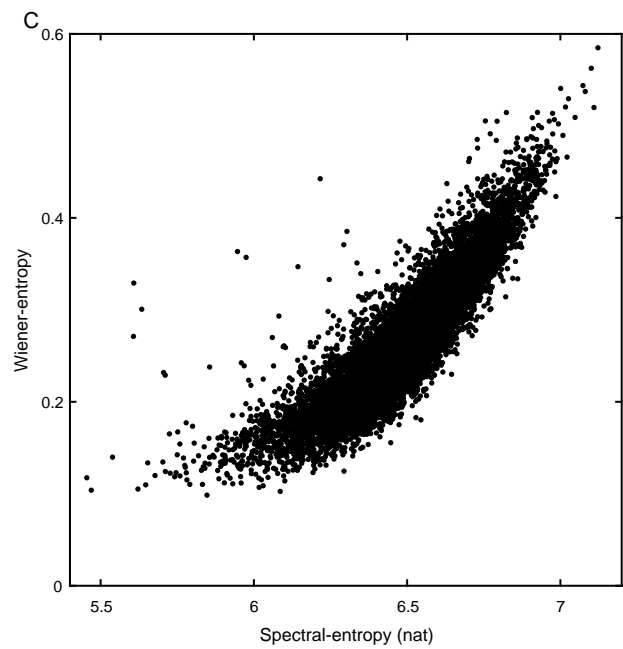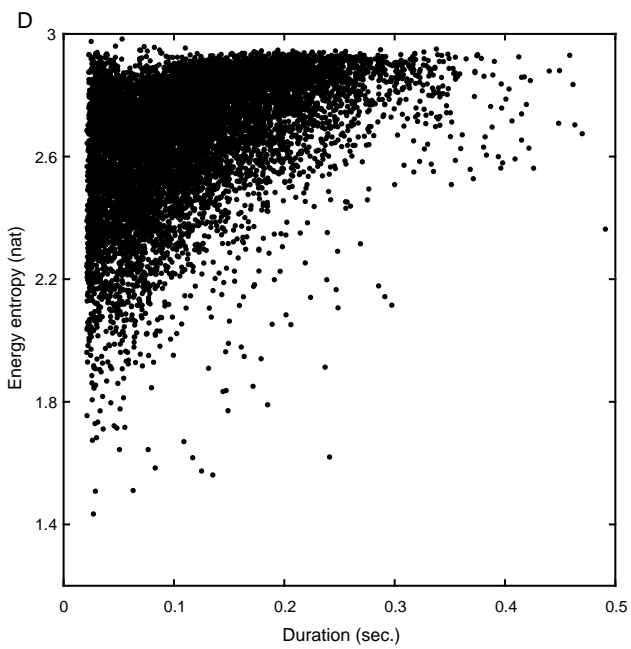

Supplement: S1 Fig — (A) Energy entropy and peak-frequency; (B) Fundamental frequency (F0) and spectral centroid; (C) Spectral entropy and Wiener entropy; (D) Duration and energy entropy. (PDF) [file pbio.2002556.s001.pdf]

**A 12-18 weeks**

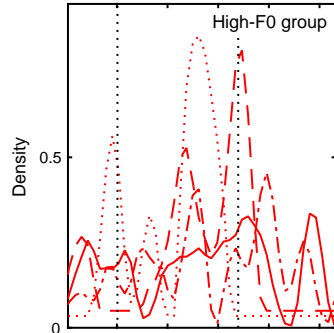

**B 31-35 weeks**

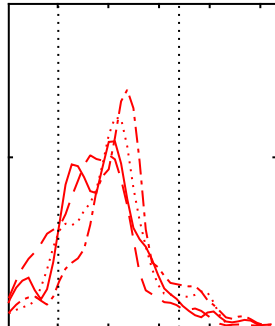

**C 40-43 weeks**

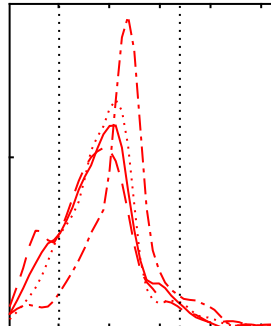

**D 48-51 weeks**

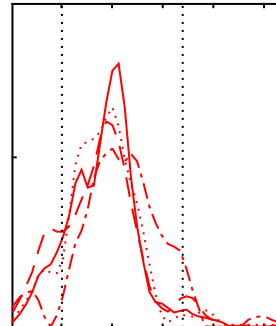

**E Control group**

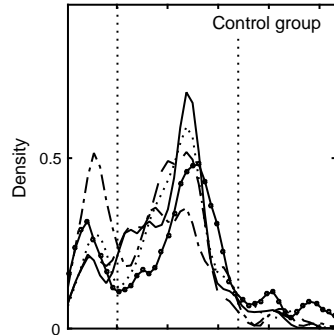

**F**

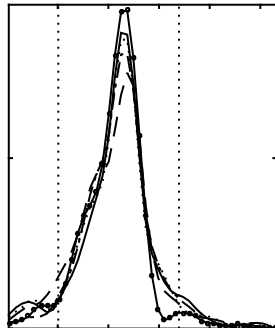

**G**

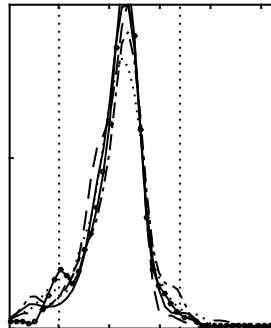

**H**

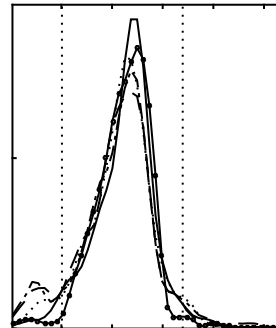

**I Low-F0 group**

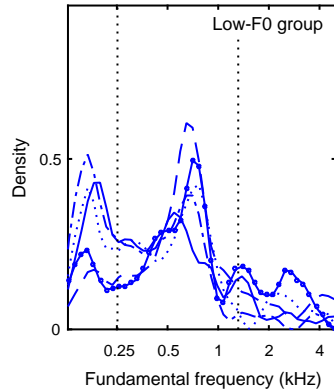

**J**

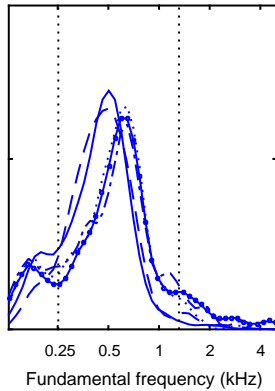

**K**

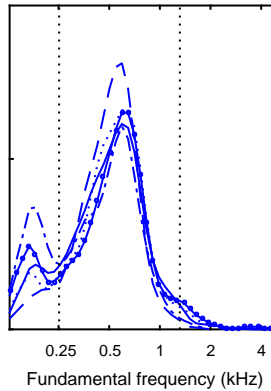

**L**

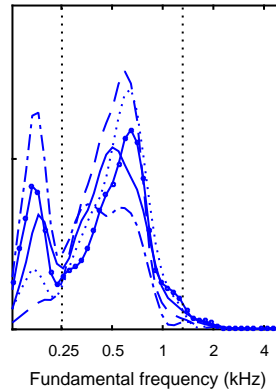

Supplement: S2 Fig — The distribution of fundamental frequency (F0) usage by each of the pups in the three groups: High-F0 (A-D), control (E-H), and Low-F0 (I-L). Each pup is plotted with a different line pattern. The four recording sessions are presented, at the ages of (A,E,I) 12–18, (B,F,J) 31–35, (C,G,K) 40–43, and (D,H,L) 48–51 weeks. (PDF) [file pbio.2002556.s002.pdf]

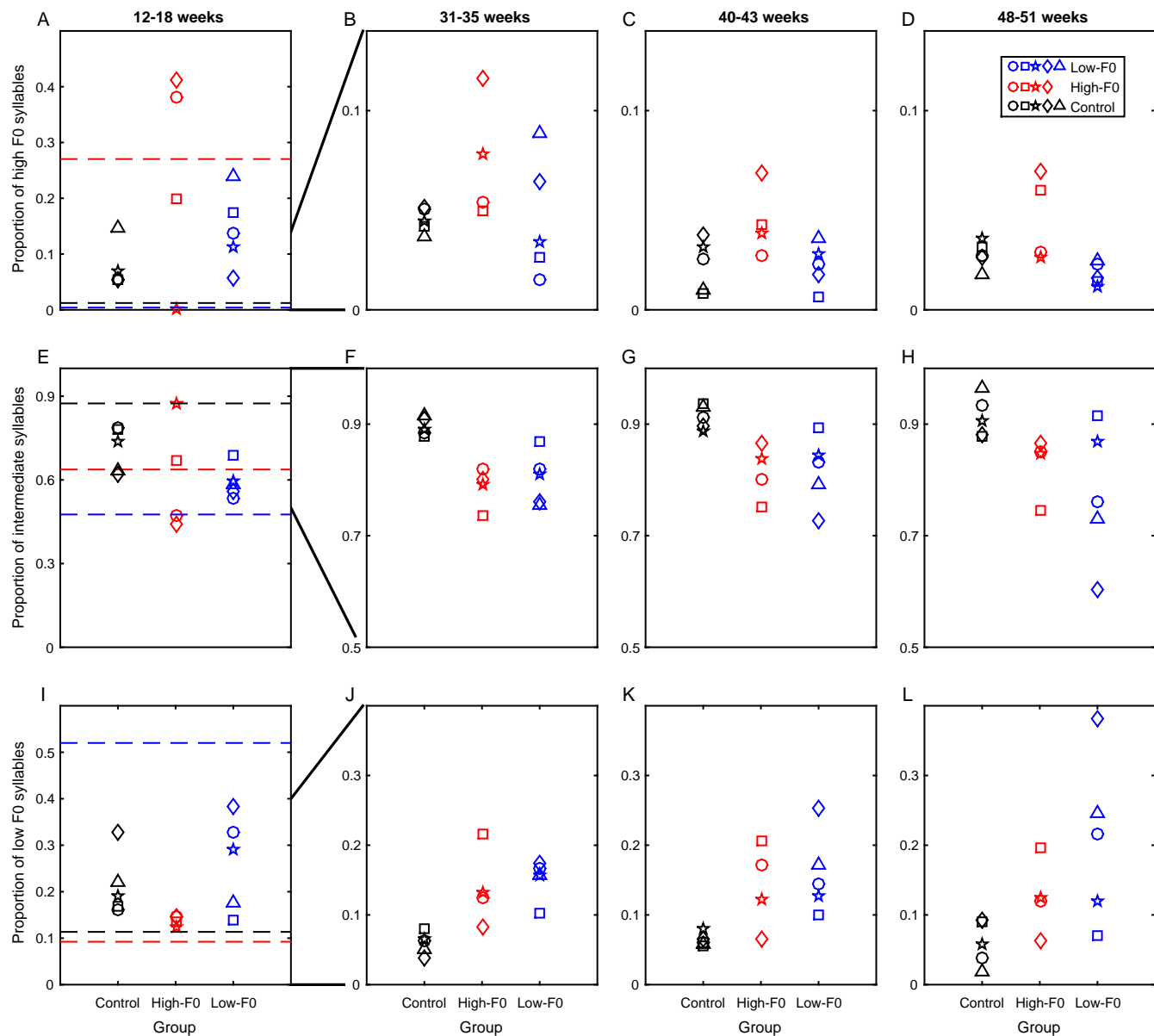

Supplement: S3 Fig — The proportion of High-F0 calls (A-D), intermediate F0 calls (E-H), and Low-F0 calls (I-L) in the vocalizations of the High-F0 group (red), Low-F0 group (blue), and control group (black). The dashed lines in (A,E,I) show the relevant proportion of calls in the playbacks of the High-F0 group (red), Low-F0 group (blue), and control group (black). The four recording sessions are presented, at the ages of (A,E,I) 12–18, (B,F,J) 31–35, (C,G,K) 40–43, and (D,H,L) 48–51 weeks. Both High-F0 and Low-F0 groups are significantly different than the control group in panels B,C,F,G,H,J,K,L One-tailed Mann-Whitney-U test, p ≤ 0.03 (see Methods for complete statistical analysis). (PDF) [file pbio.2002556.s003.pdf]

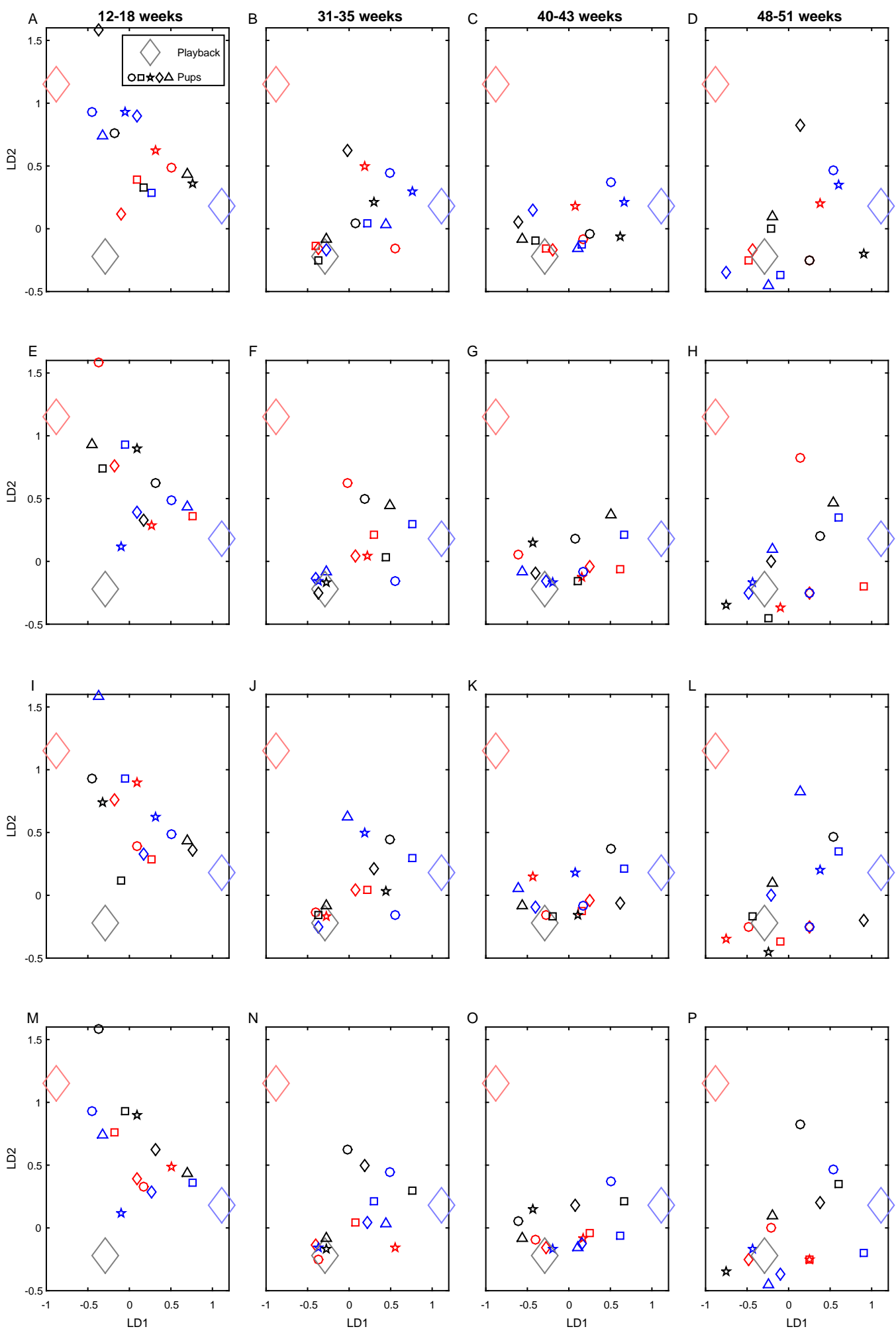

Supplement: S4 Fig — Each row is parallel to Fig 2, but the group identities of the pups were permuted. Four random permutations are shown (A-D, E-H, I-L, and M-P). The presented 4 permutations are just the first 4 that were sampled (we did not choose specific permutations), illustrating how difficult it is to get separation by chance (see text for exact p-values). The average for each bat (small symbols) and for each playback (large diamond) is presented. Blue–Low-F0 group (n = 5), red–High-F0 group (n = 4), and black–control group (n = 5). The axes were obtained by an LDA of the playbacks (see text for details). Numeric data are given in S1 Data (‘Fig 2‘ sheet)–while group identities were randomly permutated for this figure. (PDF) [file pbio.2002556.s004.pdf]

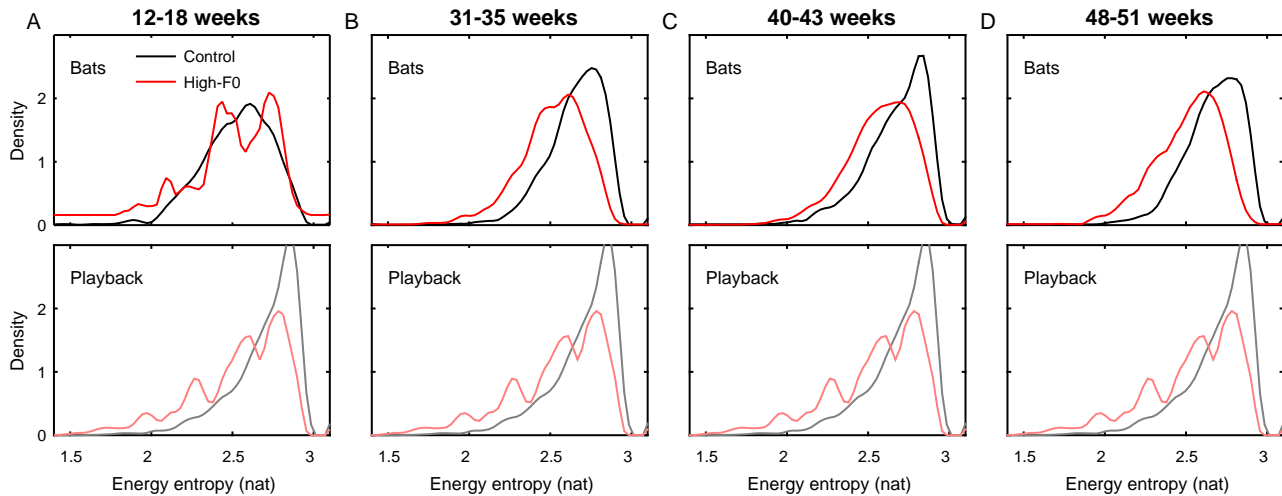

Supplement: S5 Fig — The distribution of energy entropy (Shannon entropy of the amplitude) in the High-F0 and control groups (top panels): control (black), High-F0 (red). The four recording sessions are presented as four columns: (A) 12–18 weeks, (B) 31–35 weeks, (C) 40–43 weeks, and (D) 48–51 weeks. The presented distribution is the average for all pups in the group. For comparison, the bottom panels depict the energy entropy distribution in the playback of both groups. (PDF) [file pbio.2002556.s005.pdf]
