# Supplementary material for: Crowd vocal learning induces vocal dialects in bats: Playback of conspecifics shapes fundamental frequency usage by pups
Source: PLoS Biol. 2017 Oct 31;15(10):e2002556. doi: 10.1371/journal.pbio.2002556 (PMC5663327; doi:10.1371/journal.pbio.2002556)
Supplement: S1 Table — The standardized coefficients (i.e. feature’s contribution to the discrimination in each discriminant function) and the correlation of each feature with the two linear discriminant (LD) functions. Top three are in bold. As expected by the selection of the playbacks the fundamental frequency (F0) has the major effect. The error rate in the playback discrimination was 26.4%. (PDF) [file pbio.2002556.s006.pdf]

S1 Table. Linear discriminant analysis of the playbacks.

	Energy entropy	Peak frequency	F0	Spectral centroid	Spectral entropy	Wiener entropy	Duration
Standardized Coefficients:							
LD1	**-0.592**	-0.001	**-0.857**	**-0.296**	0.194	-0.244	0.160
LD2	**-1.002**	-0.026	**0.798**	**-0.517**	0.104	0.414	0.117
Correlations:							
LD1	**-0.575**	-0.233	**-0.868**	**-0.418**	-0.062	-0.170	-0.254
LD2	**-0.753**	0.072	**0.404**	0.107	-0.018	**0.179**	-0.128

The standardized coefficients (i.e. feature's contribution to the discrimination in each discriminant function) and the correlation of each feature with the two linear discriminant (LD) functions. Top three are in bold. As expected by the selection of the playbacks the fundamental frequency (F0) has the major effect. The error rate in the playback discrimination was 26.4%.
