# Supplementary material for: Crowd vocal learning induces vocal dialects in bats: Playback of conspecifics shapes fundamental frequency usage by pups
Source: PLoS Biol. 2017 Oct 31;15(10):e2002556. doi: 10.1371/journal.pbio.2002556 (PMC5663327; doi:10.1371/journal.pbio.2002556)
Supplement: S2 Table — (PDF) [file pbio.2002556.s007.pdf]

S2 Table. Number of calls analyzed for each pup in each recording session.

Experimental group	Pup symbol (in the figures)	Recording session 1	Recording session 2	Recording session 3	Recording session 4
High-F0	Circle	34	278	476	209
High-F0	Square	15	363	702	332
High-F0	Pentagram	8	191	721	191
High-F0	Diamond	34	275	1421	143
Low-F0	Circle	110	737	1359	866
Low-F0	Square	300	600	1083	643
Low-F0	Pentagram	124	644	1115	607
Low-F0	Diamond	213	297	854	500
Low-F0	Triangle	171	589	1084	531
Control	Circle	318	1390	391	1306
Control	Square	208	502	359	285
Control	Pentagram	340	648	320	478
Control	Diamond	149	804	322	383
Control	Triangle	151	384	102	226
