# Supplementary material for: Crowd vocal learning induces vocal dialects in bats: Playback of conspecifics shapes fundamental frequency usage by pups
Source: PLoS Biol. 2017 Oct 31;15(10):e2002556. doi: 10.1371/journal.pbio.2002556 (PMC5663327; doi:10.1371/journal.pbio.2002556)
Supplement: S3 Table — Two models are presented (one for Low-F0 and one for High-F0 calls). The models included the age of the pups (four time points, i.e. recording sessions), the sex of the pups, and the group (i.e. Control, Low-F0 group, or High-F0 group). The second table of each test contains post-hoc pairwise comparisons of estimated marginal means (Bonferroni-adjusted for multiple comparisons). The p-values of factors that significantly influenced the calls are marked in bold. Insignificant interactions were removed and the models were recomputed. Analysis conducted in SPSS. (PDF) [file pbio.2002556.s008.pdf]

## S3 Table. Linear mixed models for the usage of Low-F0 and High-F0 calls.

**Tests of Fixed Effects (Proportion of Low-F0 calls):**

| Source | Numerator df | Denominator df | F | Sig. |
|---|---|---|---|---|
| **Intercept** | 1 | 51 | 79.270948 | 0.000 |
| **Group** | 2 | 51 | 9.076 | **0.000425** |
| **Sex** | 1 | 51 | .203 | .654 |
| **Age** | 1 | 51 | 11.120 | **0.001597** |

**Post-hoc pairwise comparisons (Proportion of Low-F0 calls):**

| Group/Sex 1 | Group/Sex 2 | Mean Difference | Std. Error | df | Sig. | 95% Confidence Interval | |
|---|---|---|---|---|---|---|---|
| | | | | | | Lower Bound | Upper Bound |
| ***Low-F0* group** | **Control group** | .100 | .023 | 51 | **0.0003** | .042 | .158 |
| ***High-F0* group** | **Control group** | .035 | .023 | 51 | .410 | -.023 | .094 |
| ***Low-F0* group** | ***High-F0* group** | .064 | .026 | 51 | **.049** | .000 | .129 |
| **Female** | **Male** | .009 | .021 | 51 | .654 | -.033 | .051 |

**Tests of Fixed Effects (Proportion of High-F0 calls):**

| Source | Numerator df | Denominator df | F | Sig. |
|---|---|---|---|---|
| **Intercept** | 1 | 49 | 83.982 | .000 |
| **Group** | 2 | 49 | 8.274 | **0.000802** |
| **Sex** | 1 | 49 | .719 | .400 |
| **Age** | 1 | 49 | 40.034 | **.000** |
| **Age*Group** | 2 | 49 | 4.706 | **.014** |

**Post-hoc pairwise comparisons (Proportion of High-F0 calls):**

| Group/Sex 1 | Group/Sex 2 | Mean Difference | Std. Error | df | Sig. | 95% Confidence Interval | |
|---|---|---|---|---|---|---|---|
| | | | | | | Lower Bound | Upper Bound |
| ***Low-F0* group** | **Control group** | .009 | .021 | 51 | 1.000 | -.043 | .061 |
| ***High-F0* group** | **Control group** | .063 | .021 | 51 | **.012** | .011 | .115 |
| ***Low-F0* group** | ***High-F0* group** | -.054 | .023 | 51 | .068 | -.111 | .003 |
| **Female** | **Male** | -.015 | .019 | 51 | .432 | -.052 | .023 |

Two models are presented (one for Low-F0 and one for High-F0 calls). The models included the age of the pups (four time points, i.e. recording sessions), the sex of the pups, and the group (i.e. Control, *Low-F0* group, or *High-F0* group). The second table of each test contains post-hoc pairwise comparisons of estimated marginal means (Bonferroni-adjusted for multiple comparisons). The P-values of factors that significantly influenced the calls are marked in bold. Insignificant interactions were removed and the models were recomputed. Analysis conducted in SPSS.
