# Supplementary material for: Crowd vocal learning induces vocal dialects in bats: Playback of conspecifics shapes fundamental frequency usage by pups
Source: PLoS Biol. 2017 Oct 31;15(10):e2002556. doi: 10.1371/journal.pbio.2002556 (PMC5663327; doi:10.1371/journal.pbio.2002556)
Supplement: S4 Table — For each recording session, p-values (with statistic) are depicted for Mann-Whitney-U test for differences between the sexes in high-F0 content, mean F0 production, and low-F0 content. For each recording session, p-values (with statistic) are depicted for Spearman correlation tests for correlations between body weight and high-F0 content, mean F0 production, and low-F0 content. All tests indicated insignificant relations. For each recording session, p-values (with statistic) for body-weight differences between the three experimental groups (Kruskal-Wallis test) show that there was no significant weight difference between the groups. (PDF) [file pbio.2002556.s009.pdf]

S4 Table. Similarity between males and females in F0 usage, and no correlation between body-weight and F0 usage.

Rec. session	Sex differences (Mann-Whitney-U) in: Proportion of High-F0	Mean F0	Proportion of Low-F0	Weight correlation (Spearman) with: Proportion of High-F0	Mean F0	Proportion of Low-F0	Weight differences between groups (Kruskal-Wallis)
	p-value (U)	*p-value (U)*	*p-value (U)*	*p-value (ρ)*	*p-value (ρ)*	*p-value (ρ)*	*p-value (H)*
1	0.40 (46)	0.90 (51)	0.26 (43)	0.98 (-0.01)	0.44 (-0.23)	0.26 (-0.32)	0.39 (1.87)
2	0.80 (50)	0.53 (58)	0.71 (56)	0.90 (-0.04)	0.80 (-0.08)	0.53 (-0.18)	0.37 (2.01)
3	0.32 (44)	0.45 (59)	0.21 (63)	0.99 (0.01)	0.69 (-0.12)	0.11 (-0.45)	0.29 (2.44)
4	1.00 (52)	0.80 (55)	0.07 (67)	0.60 (0.15)	0.58 (-0.16)	0.52 (-0.19)	0.27 (2.63)

For each recording session, p-values (with statistic) are depicted for Mann-Whitney-U test for differences between the sexes in high-F0 content, mean F0 production, and low-F0 content. For each recording session, p-values (with statistic) are depicted for Spearman correlation tests for correlations between body weight and high-F0 content, mean F0 production, and low-F0 content. All tests indicated insignificant relations. For each recording session, p-values (with statistic) for body-weight differences between the three experimental groups (Kruskal-Wallis test) show that there was no significant weight difference between the groups.
