# Supplementary material for: Crowd vocal learning induces vocal dialects in bats: Playback of conspecifics shapes fundamental frequency usage by pups
Source: PLoS Biol. 2017 Oct 31;15(10):e2002556. doi: 10.1371/journal.pbio.2002556 (PMC5663327; doi:10.1371/journal.pbio.2002556)
Supplement: S5 Table — * Recording rotations with only 3 pups at a time, each rotation with a different individual composition. (PDF) [file pbio.2002556.s010.pdf]

S5 Table. Recording Schedule.

Recording session	Experimental Group	Recording start	Recording end
1	Control*	21.12.15 12:45	22.12.15 12:50
	Low-F0*	22.12.15 12:50	23.12.15 12:41
	Control*	23.12.15 12:41	24.12.15 12:00
	High-F0*	27.12.15 12:50	28.12.15 12:45
	Low-F0*	28.12.15 12:45	29.12.15 13:04
	Control*	29.12.15 13:04	30.12.15 13:43
	High-F0*	30.12.15 13:43	31.12.15 14:00
	Low-F0*	03.01.16 14:34	04.01.16 14:32
	Control*	04.01.16 14:32	05.01.16 14:33
	High-F0*	05.01.16 14:33	06.01.16 15:20
	Low-F0*	06.01.16 15:20	07.01.16 15:30
	Control*	10.01.16 12:55	11.01.16 13:27
	Low-F0*	11.01.16 13:27	12.01.16 14:00
	Control*	13.01.16 11:37	14.01.16 10:00
	Low-F0*	17.01.16 13:44	18.01.16 13:17
	Control*	18.01.16 13:17	19.01.16 13:15
	Low-F0*	19.01.16 13:15	20.01.16 13:20
	Control*	20.01.16 13:20	21.01.16 14:00
	Low-F0*	24.01.16 10:40	25.01.16 11:00
	Control*	25.01.16 11:00	26.01.16 11:13
	Low-F0*	26.01.16 11:13	27.01.16 11:34
	Control*	27.01.16 11:34	28.01.16 10:00
	Low-F0*	31.01.16 10:25	01.02.16 11:20
	High-F0*	01.02.16 11:20	02.02.16 11:22
	Control	02.02.16 11:22	03.02.16 11:22
	Low-F0	03.02.16 11:22	04.02.16 11:25
	High-F0	04.02.16 11:25	05.02.16 11:50
2	High-F0	01.05.16 11:35	04.05.16 10:00
	Low-F0	05.05.16 09:40	10.05.16 10:41
	Control	10.05.16 10:41	13.05.16 11:15
	High-F0	13.05.16 11:15	16.05.16 09:51
	Low-F0	16.05.16 09:51	19.05.16 10:24
	Control	19.05.16 10:24	22.05.16 16:00
	High-F0	29.05.16 11:00	02.06.16 11:00
3	High-F0	01.07.16 09:55	07.07.16 10:53
	Low-F0	07.07.16 10:53	10.07.16 10:24
	Control	10.07.16 10:24	13.07.16 09:55
	High-F0	13.07.16 09:55	17.07.16 12:06
	Low-F0	17.07.16 12:06	20.07.16 14:42
	Control	20.07.16 14:42	25.07.16 10:00

4	High-F0	01.09.16 09:27	05.09.16 09:17
	Low-F0	05.09.16 09:17	08.09.16 09:44
	Control	08.09.16 09:44	11.09.16 09:15
	High-F0	11.09.16 09:15	15.09.16 09:30
	Low-F0	15.09.16 09:30	18.09.16 09:37
	Control	18.09.16 09:37	21.09.16 11:23
	High-F0	21.09.16 11:23	24.09.16 23:00

* Recording rotations with only 3 pups at a time, each rotation with a different individual composition
